# No Excess Mortality in Geriatric Patients With Femoral Neck Fractures Due to Shorter Intensive Care Caused by COVID-19

**DOI:** 10.7759/cureus.29986

**Published:** 2022-10-06

**Authors:** Raffael Cintean, Alexander Eickhoff, Katrin Nussbaum, Florian Gebhard, Konrad Schuetze

**Affiliations:** 1 Orthopedic Trauma Surgery, University Hospital of Ulm, Ulm, DEU

**Keywords:** intensive care unit stay, mortality, femoral neck fracture, covid-19, geriatric hip fracture

## Abstract

Background

Since March 2020, increasing numbers of hospitalized patients with coronavirus disease-2019 (COVID-19) infections have been registered. The first and the second waves necessitated the extensive restructuring of hospital infrastructure with prioritization of intensive care capacity. Elective surgeries in all surgical disciplines were postponed to preserve intensive care capacity for COVID-19 patients. However, emergency care for trauma patients had to be maintained. Especially, geriatric patients with hip fractures often require intensive care.

This study sought to investigate the possible excess mortality of geriatric patients with femoral neck fractures due to shorter intensive care unit stays because of COVID-19.

Material and methods

All patients over the age of 70 between March 2019 and February 2020 who underwent surgical treatment for femoral neck fractures were included. This cohort (group 1) was compared with all patients over 70 who received surgical treatment for hip fractures during the period of the pandemic between March 2020 and February 2021 with attention to potential excess mortality due to low intensive care capacity (group 2). Demographic data, American Society of Anesthesiologists (ASA) score, surgical modality, ICU stay, complications, and mortality were analyzed and compared.

Results

A total of 356 patients with 178 in each cohort with a mean age of 82.7 in group 1 and 84.8 in group 2 (p<0.05) were included. No significant difference was seen in sex and ASA scores. During the pandemic, patients with hip fractures had a significantly shorter stay in ICU (0.4 ± 0.9 vs 1.2 ± 2.8 days; p<0.05), shorter time to surgery (29.9 ± 8.2 vs 16.8 ± 5.3 h; p<0.05) and operations were significantly more often performed out-of-hour (4 pm-12 am 47.8% vs 56.7%; 12 am-8 am 7.9% vs 13.5%, p<0.05). Interestingly, mortality was lower during the pandemic, but the difference did not reach significance (6.7% vs 12.4%, p=0.102).

Conclusion

During the pandemic, ICU capacity was reserved for COVID patients. Due to a change in the law of the Joint Federal Committee with effect from January 1, 2021, all patients with proximal femur fractures had to be operated on within the first 24 hours, which is why a significantly shorter time to surgery was observed during the pandemic period. As a consequence, a lower mortality rate was observed, although no significance could be reached.

## Introduction

The coronavirus disease-2019 (COVID-19) pandemic caused by the severe acute respiratory syndrome coronavirus-2 (SARS-CoV-2) had a dramatic medical and economic impact worldwide. It was declared as a pandemic on March 11, 2020, by the World Health Organization (WHO), and by now it has spread to most parts of the world. By the end of November 2020, more than 62 million infected people and more than 1.4 million deaths had been reported to the WHO worldwide [1]. Due to the rapid occupancy of intensive care beds by COVID-19 patients, elective surgeries were reduced in hospitals throughout Germany and the world [2-4]. Even after a decrease in the number of infections, intensive care beds were kept free as a precaution and elective surgeries with the possible necessity of postoperative intensive care monitoring were further postponed. In Germany, up to 35% of all intensive care unit (ICU) beds were temporarily held vacant for possible COVID-19 deterioration [3].

While elective surgeries could be postponed in many departments, other surgical departments, especially trauma surgery, had to remain functional [5,6]. Various treatment methods and algorithms were developed worldwide to deal with the new circumstances in the best possible way [7-10].

Demographic changes show a rising number of the elderly population. Fragility fractures in geriatric patients increase with this trend. Additionally, these patients are often treated for chronic comorbidities, which increases the perioperative risk for severe complications and the benefit from intensive postoperative therapy. Particularly, pulmonary complications and renal failure due to pre-existing chronic failure are severe and life-limiting complications in elderly patients [11-13]. Several studies have already reported the benefits of postoperative intensive care monitoring and therapy in orthogeriatric patients [14-17]. With increasing age and the number of comorbidities, higher expectations of patients and family members, as well as extensive surgical procedures, the demand for intensive care beds is steadily increasing.

This study was designed to investigate whether increased morbidity and mortality were observed in the orthogeriatric patient group with hip fractures during the corona pandemic due to shorter stays in ICU.

## Materials and methods

The study was a retrospective exploratory review at a level one trauma center in Germany. All patients 70 years of age and older who received surgical treatment for femoral neck fractures between March 2019 and March 2021 were included in the study. All patients were treated with total hip arthroplasty, hemiarthroplasty, or osteosynthesis. The surgical modality was chosen by the attending surgeon depending on the fracture pattern, general condition, and mobility of the patient.

Patients were divided into two groups. Patients who underwent surgery between March 2019 and February 2020 were assigned to group 1, and patients who received surgical care during the time of the pandemic between March 2020 and February 2021 were assigned to group 2.

Demographic data, time of hospital admission, fracture pattern, surgical modality, length of hospital stay and length of ICU stay, comorbidities, and complications were chart reviewed and categorized. According to the group patients were assigned to, all variables were compared between groups.

Patients under the age of 70 and patients with multiple fractures as well as pathological fractures were excluded. Patients with a positive COVID-19 test on admission were excluded.

Statistics

Data analysis was performed with IBM SPSS Statistics (IBM Corp. Released 2021, IBM SPSS Statistics for Windows, Version 28.0., IBM Corp, Armonk, NY) and Microsoft Excel (Microsoft Corporation (2018), Microsoft Excel, Version 15.7). Baseline cohort characteristics were reported as means (SDs) and proportions, as appropriate, and compared using standardized mean differences to avoid identifying spurious statistical associations in the administrative dataset. Logistic regression and propensity score models were adjusted for the same covariates: age, sex, comorbidity, and surgery type.

## Results

Demographics

We found a total of 375 patients with femoral neck fractures treated with total hip arthroplasty, hemiarthroplasty, or osteosynthesis between March 2019 and March 2021. To avoid bias, 19 patients were excluded because of a positive corona test on admission. One hundred seventy-eight patients could be assigned to each group during the measured period. There was a significant difference in the age of the patients, with patients being older during the pandemic. Other variables such as gender, American Society of Anesthesiologists (ASA) classification, and Charlson comorbidity index (CCI) remained without differences (Table 1).

**Table 1:** Patient demographics. ASA: American Society of Anesthesiologists, CCI: Charlson comorbidity index. §Chi-square-test and †Mann-Whitney-test.

Variable	Total	Group 1	Group 2	p-value
Patients	356	178	178	
Mean age (years)	83.77 (70-99)	82.71 ± 8.1	84.83 ± 7.3	<0.05^§^
Male (%)	114	57 (32.0%)	57 (32.0 %)	
Female (%)	242	121 (67.9%)	121 (67.9 %)	
Mean ASA (SD)	3.0 ± 0.7	3.1 ± 0.6	3.0 ± 0.4	0.209^†^
Mean CCI (SD)	7.1 ± 1.4	6.9 ± 1.7	7.3 ± 1.1	0.252^‡^

Hospital stay

All patients were treated in an orthopedic trauma ward with orthogeriatric care. Time to surgery in group 1 was significantly longer, with an average of 29.9 hours vs 16.8 hours in group 2.

In group 1, a total of 46 patients received at least one day of postoperative intensive care. During the COVID-19 pandemic, a total of 40 patients received intensive care. No patient required preoperative intensive care. Although there was no relevant difference in the number of patients, a significant difference was found in the number of ICU days, with a longer ICU stay before the pandemic. Patients in group 1 spent an average of 1.2 ± 2.9 days in the ICU. Group 2 showed an average length of ICU treatment of 0.35 ± 0.9 days.

A cumulative total of 34 patients died within 30 days, with an overall mortality rate of 9.6%. In group 1, 22 patients died (12.4%). Although only 12 patients died in the COVID group (6.7%), no significance could be reached (Table 2).

**Table 2:** Characteristics of patients after a femoral neck fracture treatment. §Chi-square-test, *minimum one.

Variable	Total	Group 1	Group 2	p-value
Mean time-to-surgery (hours, SD)	23.4 ± 53.9	29.9 ± 69.9	16.8 ± 29.0	<0.05^§^
Time of surgery				
During office hours (n)	132	79	53	0.117^§^
After hours (n)	224	99	125	<0.05^§^
Mean ICU stay (days, SD)	0.8 ± 1.4	1.2 ± 2.9	0.35 ± 0.9	<0.05^§^
Complications* (n)	116 (32.6%)	63 (35.4%)	53 (29.8%)	0.893^§^
Thirty-day mortality (n, %)	34 (9.6%)	22 (12.4%)	12 (6.7%)	0.103^§^

Logistic regression was performed to adjust for differences in surgical modality, preoperative health status, and age of patients. Groups, patient age, ASA less than three, equal to or greater than three, and different surgical modalities were compared. This showed a 3.6-fold higher likelihood of receiving at least one day of ICU care if patients had surgical treatment prior to the pandemic independent of other variables. No significant associations could be found in age, preconditions or surgical modalities (Table 3).

**Table 3:** Logistic regression model ASA: American Society of Anesthesiologists, β-reg: beta-regression, SE: standard error, OR: odds ratio.

Variable	β-reg	SE	Wald test	p-value	OR
Group (1, 2)	−1.273	0.394	10.458	0.001	0.280
ASA (<3, >=3)	1.173	0.752	2.433	0.119	3.232
Age	0.013	0.022	0.333	0.564	1.013
Surgical modality	−0.266	0.455	0.341	0.559	0.767

**Figure 1:**
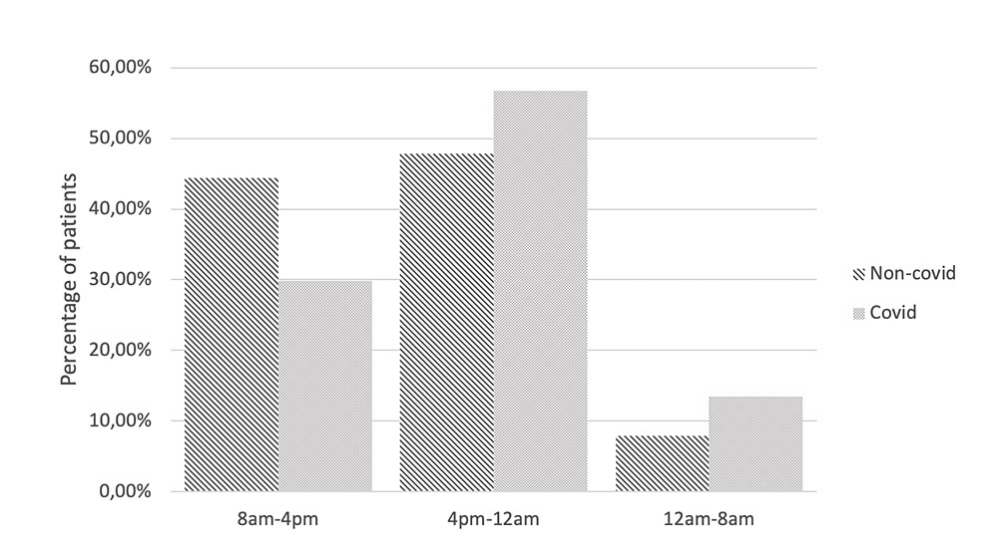
Time distribution of surgical treatment of patients with hip fractures before and during the corona pandemic. Group 1: non-COVID, Group 2: COVID.

Patients were significantly more likely to get surgical treatment during office hours prior to the pandemic. It was found that 44.4% of all patients with hip fractures underwent surgery between 8 am and 4 pm. Only 7.9% of patients were operated on between 12 am and 8 am. During the pandemic, significantly more patients were treated in the after-hours (p<0.05). About 56.7% of all patients underwent surgery between 4 pm and 12 am, and as many as 13.5% of all patients between 12 am and 8 am (Figure 1).

Complications

**Figure 2:**
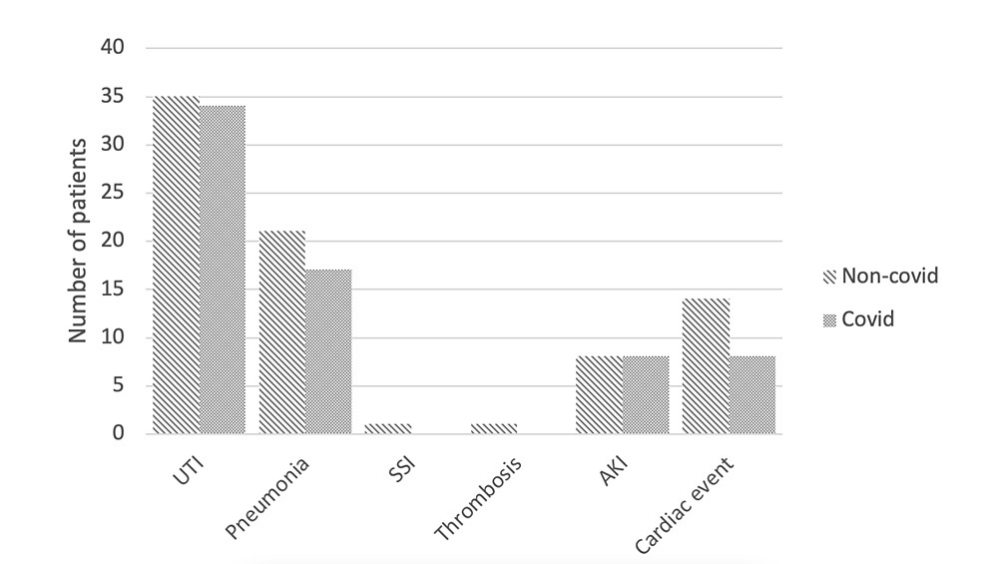
Complications during the stay. UTI: urinary tract infection, AKI: acute kidney injury, SSI: surgical site infection, Group 1: non-COVID, and Group 2: COVID.

We found a total of 147 complications during the inpatient stay, with 116 patients suffering from at least one complication. Patients suffered most frequently from urinary tract infections (UTI) as well as non-COVID-19 pneumonia, followed by cardiac events and acute kidney injury (AKI). One patient suffered from an early surgical site infection (SSI) and another one from deep vein thrombosis. No significant difference in complications between the groups could be found (Figure 2).

Mortality

A total of 34 patients (9.6%) died in the first 30 days after surgery. Twenty-two patients (12.4%) died in group 1 between March 2019 and February 2020, while 12 patients (6.7%) died in group 2 between March 2020 and February 2021.

In group 1, the main causes of death were acute decompensated heart failure (40.9%), pneumonia (27.2%), and acute chronic renal failure (18.2%).

During the pandemic, all patients received a polymerase chain reaction (PCR) swab test on admission, whereas 19 patients tested positive (10.7%). Eight patients died from non-COVID-19-related pneumonia (66.6%), three from decompensated heart failure (25%), and one from acute chronic renal failure (8.4%).

## Discussion

The present study investigated the impact of shorter ICU stays due to the COVID-19 pandemic on geriatric patients with femoral neck fractures. Contrary to our expectations, we found lower mortality during the pandemic, although patients received significantly shorter periods of intensive care.

It is already widely known that hip fractures in elderly patients are associated with increased mortality. Early, postoperative mortality after hip fracture in geriatric patients is seen in studies as an indirect indicator of the quality of care [18-20]. Blanco et al. reported a 30-day mortality of 6.0% in a cohort of 923 patients with a significantly higher mortality rate in the age group above 90 years [18]. In a study with 467 patients, Khan et al. found a 30-day mortality rate of 7.5% [20]. Other studies reported similar mortality rates within the first 30 days in comparable patient populations [19,21-23]. In the present study, we found an overall 30-day mortality rate of 9.6% with a higher 30-day mortality rate of 12.4% in the pre-corona pandemic patient group in particular.

In a large cohort study of over 160,000 patients, cardiovascular disease as a result of trauma was found to be a leading cause of death in geriatric patients with hip fractures [24]. Other studies report similar results [13,25]. We found comparable results in our group of patients before the corona pandemic, where cardiovascular diseases were identified as the main cause of death.

Surprisingly, the cohort during the corona pandemic showed lower mortality, contradicting the current literature. In the present study, we found a mortality rate of 6.7% in the period between March 2020 and February 2021. Egol et al. found a significant increase in mortality with an overall rate of 12.3% in geriatric patients with hip fractures during the corona pandemic. In their study, 17 patients with acute COVID-19 infection were included, showing a 30-day mortality rate of 52.9% [7]. Walters et al. reported no significant change in mortality rates among patients with hip fractures during the corona pandemic with a rate of 5.8%, with the time period from March 2020 to May 2020 covering only the first wave of infection [26]. All studies on hip fractures in geriatric patients during the corona pandemic reported that respiratory infections were predominant for a prolonged stay, increased postoperative complications, or increased mortality [7,8,18,26]. We found similar results. While cardiovascular causes were leading prior to Corona, both COVID-19-associated pneumonia and pneumonia caused by other pathogens were shown to be major causes of death, with COVID-related mortality of 33.3%.

One possible explanation for the decreased mortality rate during the corona pandemic in the present study is the significantly shorter time to surgery, which was an average of 16.8 hours. Since January 1, 2021, it has been decided by the Joint Federal Committee in Germany that proximal femur fractures must generally be operated on within the first 24 hours [27]. In addition, various standard operating procedures (SOP) were required. These provide guidelines for the preoperative management of patients and, in particular, surgical preparation of geriatric patients. Here, for example, the handling of anticoagulants and the antagonization of these are specified. Pincus et al. already showed in their 2017 study that time to surgery over 24 hours can be unfavorable to survival [21]. Simunovic et al. provided similar results in their systematic review of 16 studies with over 13,000 patients with shorter time to surgery being associated with a reduced risk of pneumonia and mortality [28]. In addition to the complications and increased mortality due to delays in surgical care, Ogawa et al. showed that surgery within the first 24 hours of arrival was associated with significantly improved postoperative mobility [29].

Studies report shorter times for surgeries during the pandemic as a result of increased operating room capacity, which is explained by the suspension of elective surgeries [6,8]. In our experience, although elective surgeries were postponed, the availability of operating rooms was reduced by shifting personnel to the intensive care unit. As a result, surgeries were distributed throughout the day and performed significantly more often out-of-hours during the corona pandemic, which resulted in a significant reduction in time-to-surgery. Additionally, this may be due to the not inconsiderable deductions of reimbursement by health insurance companies if surgeries are performed with a delay of more than 24 hours without a medical reason.

The reduced time to surgery and consecutive reduction in morbidity and mortality could also be the reason for the shortened time of ICU stay in the present study. Patients treated within the time period of the COVID pandemic were significantly shorter in the ICU compared to patients before the pandemic. Eschbach et al. reported in their 2016 study the mean ICU stay of geriatric patients with hip fractures of 2.5 days [14]. We found an ICU stay of 1.2 days on average pre-COVID with a reduction to 0.35 days on average during the pandemic. Hasan et al. reported no significant prolongation in ICU treatment due to surgical delay over 48 h, although mentioning that patients with a higher CCI are more likely to need intensive medical care [30]. Comparable studies related to the corona pandemic and the necessity of intensive postoperative therapy could not be found in the literature. Our assumption is, on one hand, that ICU beds were reserved for COVID patients and the threshold for ICU admission was raised due to high demand because of COVID patients. On the other hand, based on the new guidelines, preoperative treatments were intensified, and as a conclusion, patients showed a better postoperative outcome and less necessity for intensive care.

Limitations

Due to its retrospective nature, some of the data may not be accurate. It is a single-center study, which limits the number of patients and provides data acquisition bias. Due to the limited time frame, long-term results were unavailable to obtain and may be unrepresented.

## Conclusions

The pandemic puts high pressure on medical infrastructure as well as personnel. Despite that, especially the vulnerable group of orthogeriatric patients, they were still in need of medical care and treatment. In our study, we showed that geriatric patients with femoral neck fractures were less likely to receive postoperative intensive care with shorter ICU stays during the corona pandemic. In addition, they received significantly more often out-of-hours surgical care, which, however, resulted in a shorter time to surgery. Regardless of age, pre-existing conditions, and type of surgical care, geriatric patients with femoral neck fractures showed lower 30-day mortality compared with the pre-pandemic period. We recommend early surgical treatment of hip fractures in geriatric patients, without neglecting thorough preoperative preparation. 
